# A Multilayer Secure Biomedical Data Management System for Remotely Managing a Very Large Number of Diverse Personal Healthcare Devices

**DOI:** 10.1155/2015/941053

**Published:** 2015-07-13

**Authors:** KeeHyun Park, SeungHyeon Lim

**Affiliations:** Keimyung University, Daegu 704-701, Republic of Korea

## Abstract

In this paper, a multilayer secure biomedical data management system for managing a very large number of diverse personal health devices is proposed. The system has the following characteristics: the system supports international standard communication protocols to achieve interoperability. The system is integrated in the sense that both a PHD communication system and a remote PHD management system work together as a single system. Finally, the system proposed in this paper provides user/message authentication processes to securely transmit biomedical data measured by PHDs based on the concept of a biomedical signature. Some experiments, including the stress test, have been conducted to show that the system proposed/constructed in this study performs very well even when a very large number of PHDs are used. For a stress test, up to 1,200 threads are made to represent the same number of PHD agents. The loss ratio of the ISO/IEEE 11073 messages in the normal system is as high as 14% when 1,200 PHD agents are connected. On the other hand, no message loss occurs in the multilayered system proposed in this study, which demonstrates the superiority of the multilayered system to the normal system with regard to heavy traffic.

## 1. Introduction

As awareness and interest in health issues have become widespread and the number of elderly persons has grown over recent years, the concept of healthcare has shifted from disease diagnosis and treatment to disease prevention. As a result, the utilization of Personal Healthcare Devices (PHDs) has increased substantially [1–8]. Moreover, due to the rapid technical development of various systems and the advent of the ubiquitous era, it is expected that PHD utilization will continue to increase.

However, the proliferation of PHDs will bring with it a new problem, effective remote device management for a very large number of PHDs. As one of several types of mobile healthcare devices, PHDs need to be managed closely and accurately. There are many reasons to have PHDs managed by systems or healthcare organizations. First of all, PHDs are healthcare devices, and poor management of PHDs could cause very serious healthcare problems. Secondly, most PHD users are elderly patients who may have problems handling their PHDs skillfully. Therefore, it is most appropriate for healthcare organizations or healthcare systems to take care of PHD management work for PHD users. However, when the number of PHDs to be managed is very large, another problem occurs. This is the fact that there would most likely be a bottleneck problem [9, 10] in a PHD management system, which in turn would cause a serious performance problem. In addition, whenever biomedical data is processed, security issues related to the data inevitably arise. The biomedical data of a user must be securely protected and cannot be accessed/altered by an unauthorized person, as this is a serious invasion of privacy.

Therefore, in this paper, multilayer secure biomedical data management architecture for managing a very large number of diverse personal health devices is proposed. The system has the following characteristics: first, the system is designed in a hierarchical fashion to lessen the bottleneck problem that might be caused by requests from a very large number of diverse PHDs. Second, the system supports international standard communication protocols to achieve interoperability. Two protocols, ISO/IEEE 11073 protocol and OMA DM (device management) protocol, which were proposed as international standard communication protocols for PHD communication and remote device management, respectively, are extended and implemented in the system. Third, the system is integrated in the sense that both a PHD communication system and a remote PHD management system work together as a single system. Finally, the system proposed in this paper provides user/message authentication processes to securely transmit biomedical data measured by PHDs based on the concept of a biomedical signature.

Some experiments, including the stress test, were conducted to show that the system proposed in this study performs very well even when a very large number of PHDs are used. Moreover, after locating the bottlenecks, it was found that there was still room to improve the system performance, and two modifications of the system were performed in this study, database division and delayed write. It is found that the system performance was much improved after these two modifications were applied.

Moreover, message loss is considered to be a very serious issue in health related systems such as the one discussed in this study. Through the experiments, it is found that the loss ratio of the ISO/IEEE 11073 messages in the normal system (e.g., the system without any gateways) is quite high in heavy traffic, while no message loss occurs in the multilayered system proposed in this study, which demonstrates the superiority of the multilayered system to the normal system in regard to heavy traffic.

The remainder of this paper is organized as follows.  Section 2 describes some related studies, Section 3 explains the multilayered remote PHD management system for a very large number of PHDs proposed in this paper, Section 4 discusses security considerations for the system, and Section 5 shows the results of some experiments using the system constructed in this study, along with a discussion based on the results. In addition, two modification schemes to improve performance are explained. Finally, Section 6 draws some conclusions and discusses some possible directions for future research.

## 2. Related Studies

### 2.1. ISO/IEEE Communication Protocol

**Figure 1:**
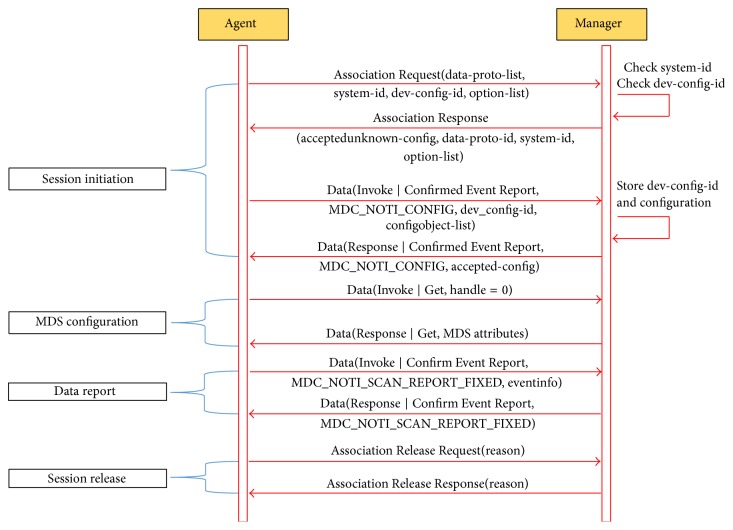
ISO/IEEE 11073 communication protocol sequence.

The ISO/IEEE 11073 communication protocol [3–5] was proposed by an ISO/IEEE committee as an international standard to provide interoperability for health and medical services in ubiquitous environments (especially using PHDs). PHDs are small-sized healthcare devices which can be used at home without the direct intervention of medical personnel. Examples of PHDs are physical activity monitors, blood pressure monitors, glucose meters, and medication dispensers [3–6, 8]. A PHD-related system defined by ISO/IEEE consists of agents and managers. An agent is a program installed in the PHD. A manager is a program installed in a server (e.g., a laptop computer or a PC) which receives/processes biomedical data from PHDs to provide a user's health information to medical personnel. The sequence of the ISO/IEEE 11073 communication protocol is shown in Figure 1 [3]. The sequence of the protocol consists of session establishment (“Association Request” and “Association Response” in the figure), data transmission, and session release (“Association Release Request” and “OMA DM communication protocol Association Release Response” in the figure).

### 2.2. OMA DM Communication Protocol

**Figure 2:**
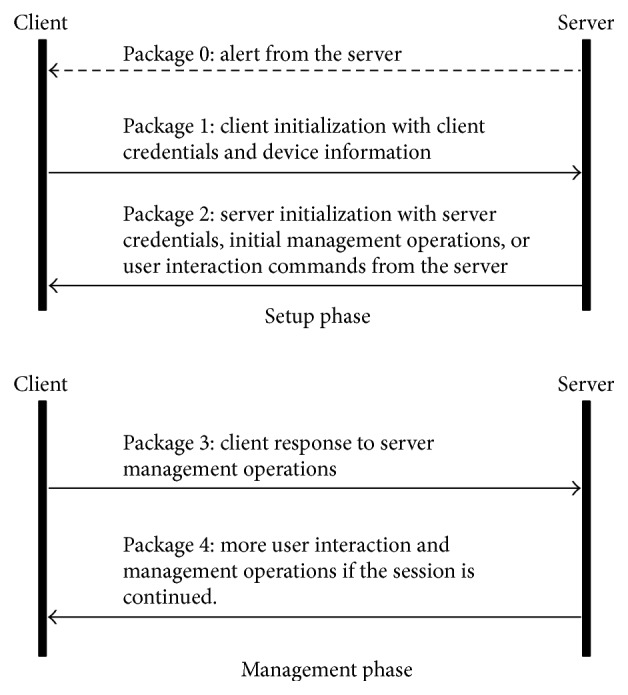
OMA DM communication protocol sequence.

The OMA DM communication protocol was proposed by OMA (Open Mobile Alliance) as an international standard for the remote management of mobile devices [11]. Since then, the protocol has become used very widely [12–15]. However, attempts to apply the protocol to manage PHDs have been rare. An OMA DM-related system consists of clients (DM agents) and servers (DM managers). A DM agent, installed in a mobile device, executes device management operations issued by a DM manager. Objects managed by DM agents or DM managers are grouped together to form DM trees, whose nodes are called management objects. The sequence of the original OMA DM communication protocol is shown in Figure 2 [11]. The sequence of the protocol consists of a setup phase and a management phase. In the setup phase, authentication and device (agent) information are exchanged between a DM agent and a DM manager. In the management phase, management commands and their status reports are exchanged.

### 2.3. Integrated Gateway for PHDs

**Figure 3:**
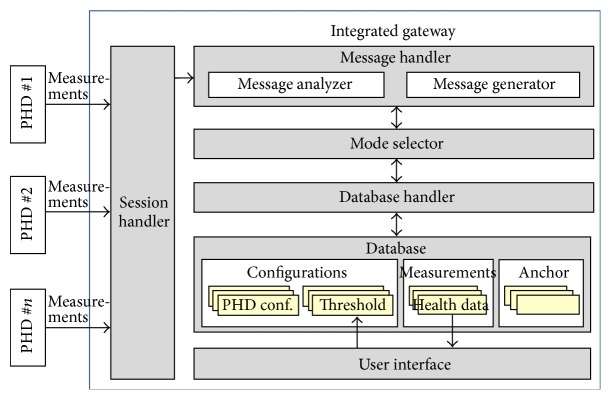
Structure of the integrated gateway in [16].

In [16], an integrated gateway for diverse PHDs was proposed, as shown in Figure 3. This gateway receives measurements from diverse PHDs and conveys them to a remote monitoring server. It provides two kinds of transmission modes: immediate transmission and integrated transmission. The former mode operates if a measurement exceeds a predetermined threshold, or in the event of an emergency. In the latter mode, the gateway retains the measurements instead of forwarding them. When the reporting time comes, the gateway extracts all the stored measurements, integrates them into one message, and transmits the integrated message to the monitoring server. In this study, only three PHDs (e.g., an activity monitor, a medication dispenser, and a pulse oximeter) were used, and therefore there is no need to be concerned about the bottleneck problems that can create severe degradations in system performance. Furthermore, the study did not work on remote PHD management.

In [17], a message processing scheme for an integrated PHD gateway in an integrated PHD management system that serves diverse PHDs is proposed. On receiving the ISO/IEEE-based health messages generated by PHDs, the integrated PHD gateway performs some integration work to send an integrated message to the integrated PHD management server. Also, when the integrated PHD gateway receives the OMA DM-based PHD management messages from the server, the gateway performs some separation work to send the messages to the related PHDs separately.

Located between PHDs and the integrated PHD management server, integrated PHD gateways transform the ISO/IEEE 11073 based messages into OMA DM based messages and vice versa. The ISO/IEEE 11073 communication protocol is used to transmit health messages measured by a PHD to the integrated PHD management server via the related integrated PHD gateway. The OMA DM communication protocol is used to transmit device management commands issued by the integrated PHD management server to a PHD via the related integrated PHD gateway. This study also used only three PHDs, and therefore there is no need to be concerned about the bottleneck problems that can create severe degradations in system performance. Therefore, the system proposed in [15] may not be used in a scenario in which there are a very large number of PHDs. A single gateway system was used in this study.

## 3. Secure Biomedical Data Management System

### 3.1. System Overview

**Figure 4:**
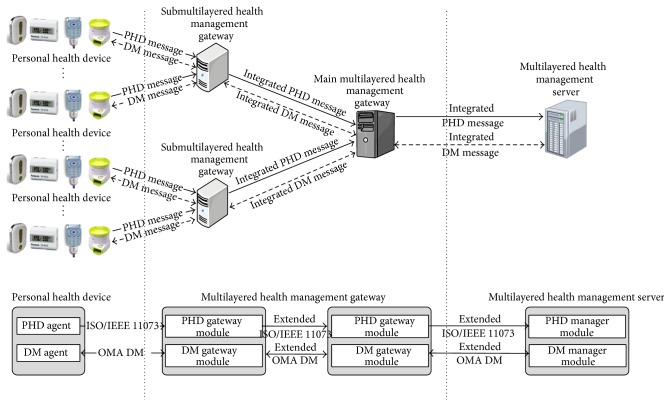
Structure of secure biomedical data management system with a very large number of PHDs.

Figure 4 shows the secure biomedical data management system for a very large number of PHDs that is proposed in this paper. The secure biomedical management system has the following components:

- PHD and PHD agent: as defined by ISO/IEEE 11073, a PHD agent, installed in a PHD, captures a user's biomedical signals, processes the signals to form health data, and sends the data to the multilayered health information server, via the multilayered health management gateways related to the PHD. In addition, the PHD agent executes remote management commands issued by the multilayered health information management server via the gateways. Then, the PHD agent sends status reports to the server via the gateways. The health data and health information are delivered in ISO/IEEE 11073 messages, while the management commands and status reports are delivered in OMA DM messages. In this study, PHD agents can handle not only ISO/IEEE 11073 messages but also OMA DM messages.
- Multilayered health management gateways and their programs: these gateways receive health data or status reports from PHD agents and process them to produce health/management information in order to send the information to the server. In addition, they receive remote management commands to send to the PHD agents from the server. To reduce communication traffic, the gateways do not try to send the data as soon as the data arrives. The gateways accumulate the data from the PHD agents for a while to integrate the data in order to form a single (extended) ISO/IEEE 11073 or OMA DM message. Also, the gateways disintegrate a single (extended) ISO/IEEE 11073 or OMA DM message, received from the server, to distribute separate messages to individual PHD agents. In Figure 4, a 2-layered gateway system is presented, but the system is designed and constructed to support more than 2 layers of the gateways to distribute heavy traffic loads over the entire system when the number of active PHD agents at once is very large. In this study, the ISO/IEEE 11073 protocol is extended in the sense that the concept of the gateway is added to service a very large number of PHDs without causing a severe degradation in system performance.
- Multilayered health management server and its programs: this server receives health information/status reports from the PHD agents via the gateways and processes them for medical personnel. The server also issues remote management commands for PHD agents. The management commands implemented in this study are ADD, REPLACE, and DELETE.

### 3.2. PHD Agent

**Figure 5:**
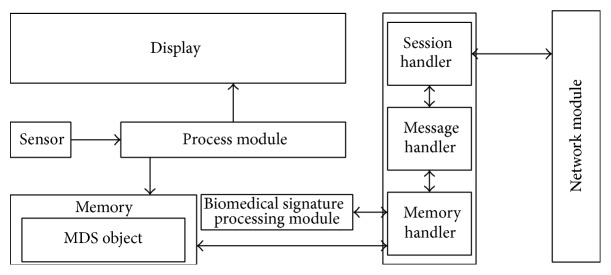
Structure of the PHD and the PHD agent.

As mentioned earlier, the PHD agent in the PHD captures a user's health data to send to the server via gateways. As shown in Figure 5, the PHD agent consists of a session handler, a message handler, a memory handler, and a biomedical signature processing module.

- Session handler establishes/releases a communication session between a PHD agent and a gateway to which the agent is connected, using the network module in the PHD.
- Message handler creates ISO/IEEE 11073 messages using health data stored in the memory of the PHD and sends the messages to the session handler.
- Memory handler accesses health data from the memory of the PHD for the message handler. It also stores new health data in the memory.
- Biomedical signature processing module generates a hash value based on new biomedical data of a user measured by a PHD. The hash value will be used by the health management server to authenticate the new biomedical data.

**Figure 6:**
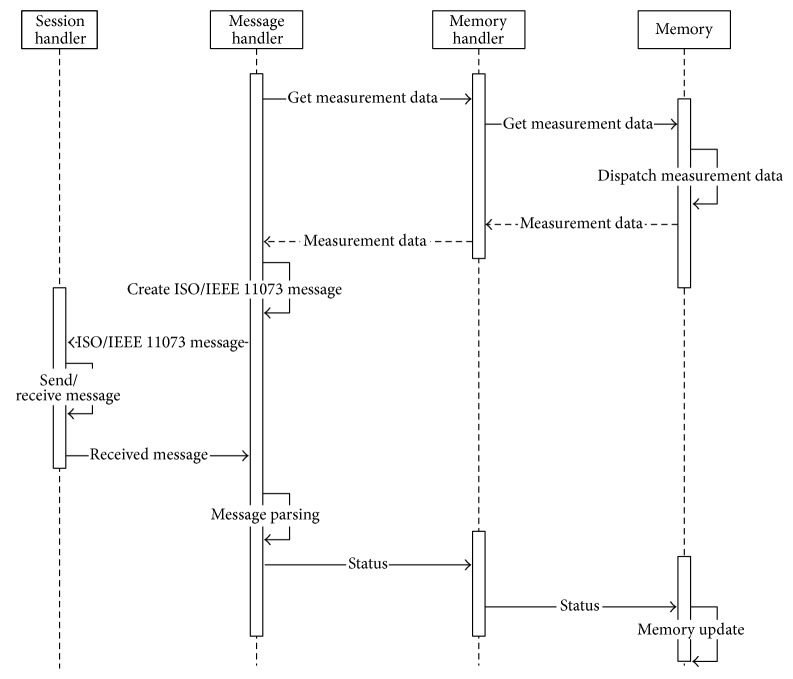
Sequence diagram of messages in a PHD agent.

Figure 6 shows the sequence diagram of messages in a PHD agent.

### 3.3. DM Agent

**Figure 7:**
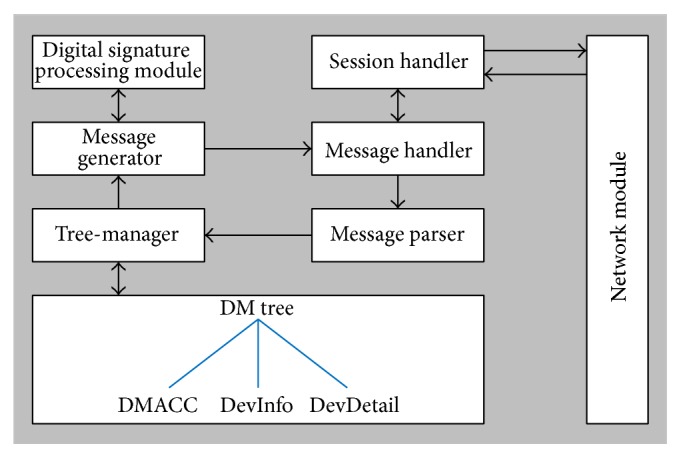
Structure of the DM agent.

Figure 7 shows the structure of the DM agent of the PHD. The DM agent receives remote management commands from the server via the gateways, executes the commands, and sends status reports to the server. The DM agent consists of a session handler, a message handler, a message parser, a message generator, and a tree-manager.

**Figure 8:**
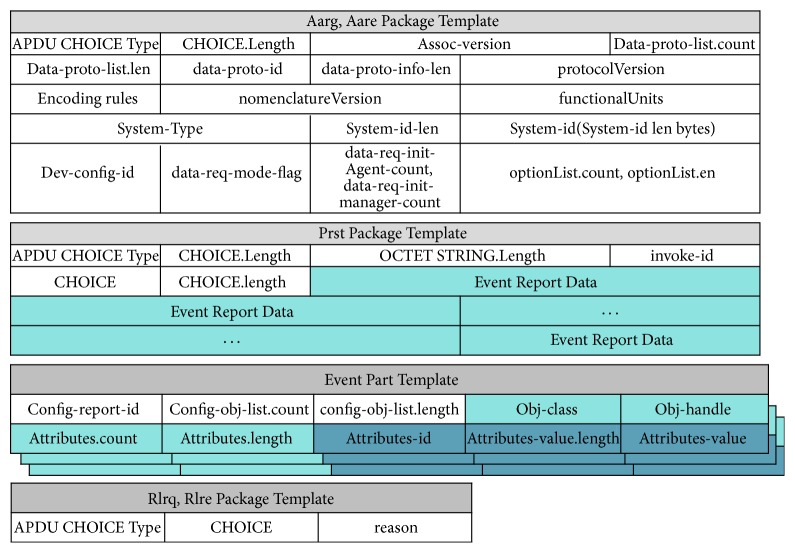
Structure of package templates.

- Session handler is similar to the session handler of the PHD agent.
- Message handler receives OMA DM messages from the session handler to execute remote management commands.
- Memory handler accesses a DM tree from the memory of the PHD for the message handler. It also stores an updated DM tree in the memory.
- Message parser receives OMA DM messages from the message handler and parses the messages to extract remote management commands to execute.
- Tree-manager updates the DM tree in the DM agent according to the results of the remote command execution.
- Message generator generates OMA DM messages to include the results/status of the remote command execution. The generated OMA DM messages (or packages) are sent to the server.  Figure 8 shows the structure of the package templates for OMA DM messages. All of the fields except for the dark colored fields remain unchanged during the session. Therefore, when a message is generated, it uses a template to reduce message generation time.

**Figure 9:**
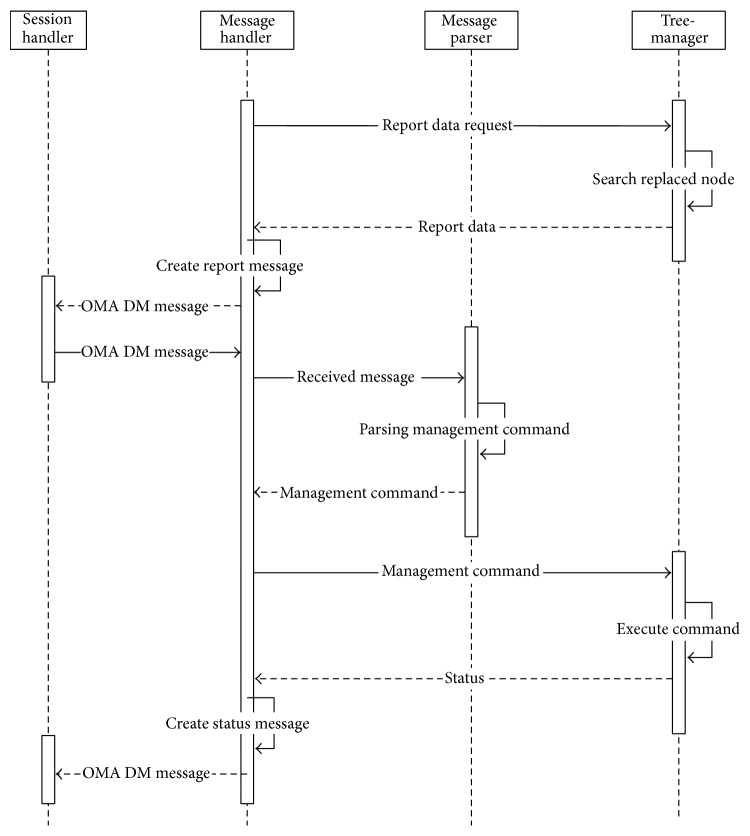
Sequence diagram of messages in a DM agent.

Figure 9 shows the sequence diagram of messages in a DM agent.

### 3.4. Multilayered Health Management Gateway

**Figure 10:**
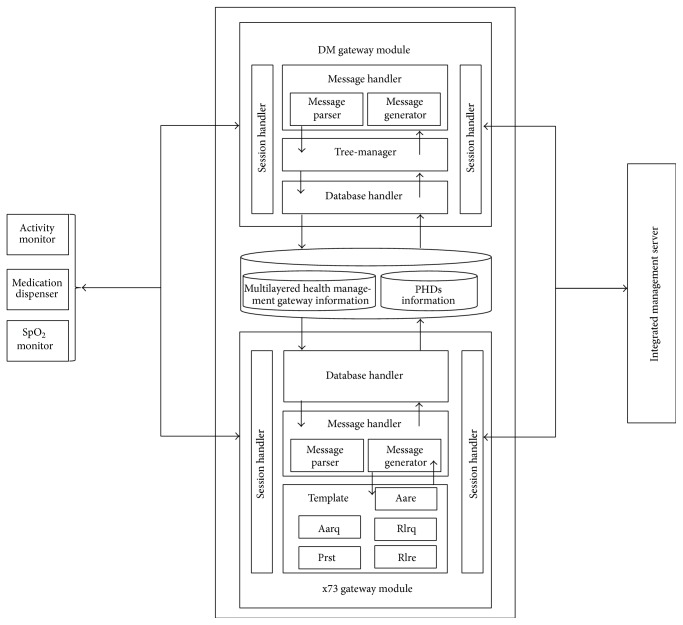
Structure of the multilayered health management gateway.

Figure 10 shows the structure of the multilayered health management gateway. The gateway consists of 2 modules: a PHD gateway module and a DM gateway module.

#### 3.4.1. PHD Gateway Module

The PHD gateway module receives separate ISO/IEEE 11073 messages from the PHD agents of the PHDs. The gateway updates its own database to store measured health data and integrates the data into a single integrated message to send to the gateway in the higher layer or the server. On the other hand, when the gateway module receives an integrated ISO/IEEE 11073 messages from the gateway in the higher layer or the server, the gateway module updates its own database and disintegrates the message into several separate ISO/IEEE 11073 messages to send to PHDs or the gateways in the lower layer. The gateway module consists of a session handler, a message handler, and a database handler.

- Session handler controls sessions between the PHD and the gateway in the higher layer or the server.
- Message handler processes ISO/IEEE 11073 messages received from the PHD or the gateway in the lower layer or the server and generates an integrated ISO/IEEE 11073 message to send to the gateway in the higher layer or the server. It is important to note that the messages transmitted have many parts in common. Therefore, when a message is generated, it uses a template to reduce message generation time.
- Database handler accesses/stores health data from/to the database. The gateway identifies the MDS (Medical Device System) configuration of the connected PHD by examining the Configuration ID and the System ID, which specifies the characteristics of the PHD. According to the MDS configuration, the received health data is classified in the database.

**Figure 11:**
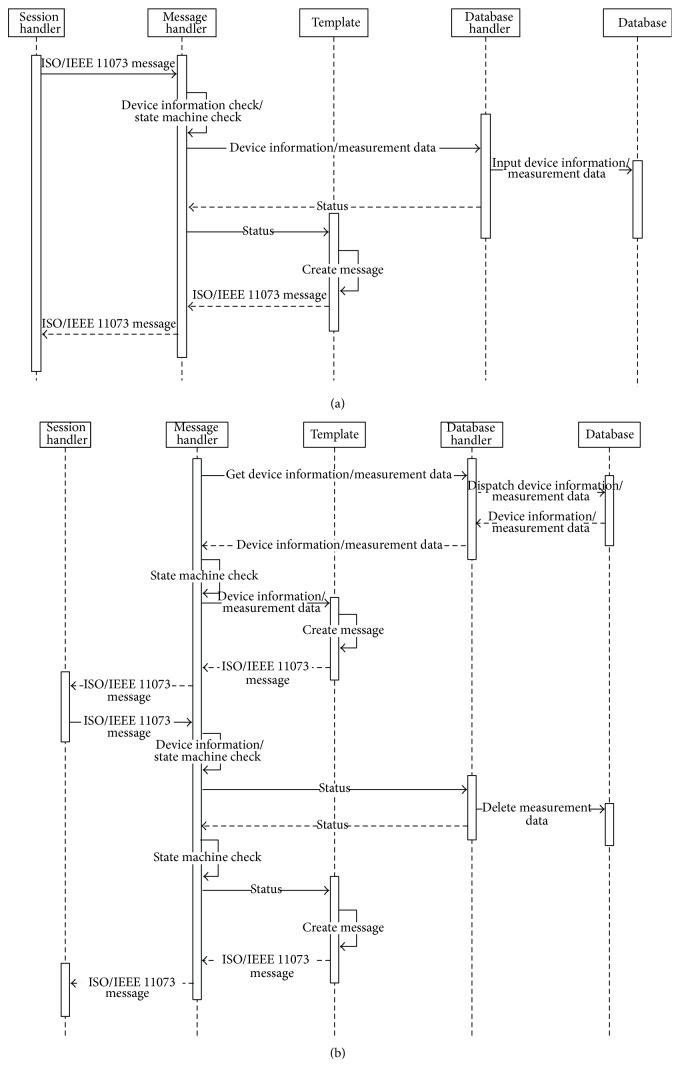
(a) Sequence diagrams of message reception in a PHD gateway. (b) Sequence diagrams of message integration in a PHD gateway.

Figures 11(a) and 11(b) show the sequence diagrams of message reception (a) and message integration (b) in a PHD gateway.

#### 3.4.2. DM Gateway Module

Upon receiving the separate OMA DM messages from the PHDs or the gateways in the lower layer, the DM gateway module integrates the received messages into a single integrated message, updates its own DM tree, and sends the message to the gateway in the higher layer or the server. On the other hand, when the DM gateway module receives an integrated OMA DM message from the gateway in the higher layer or the server, the gateway module updates its own DM tree and disintegrates the message into several separate OMA DM messages to send to PHDs or the gateways in the lower layer. The gateway module consists of a session handler, a tree-manager, and a database handler. The components of the DM gateway module are very similar to those of the DM agent, and thus an explanation of these will be omitted.

**Figure 12:**
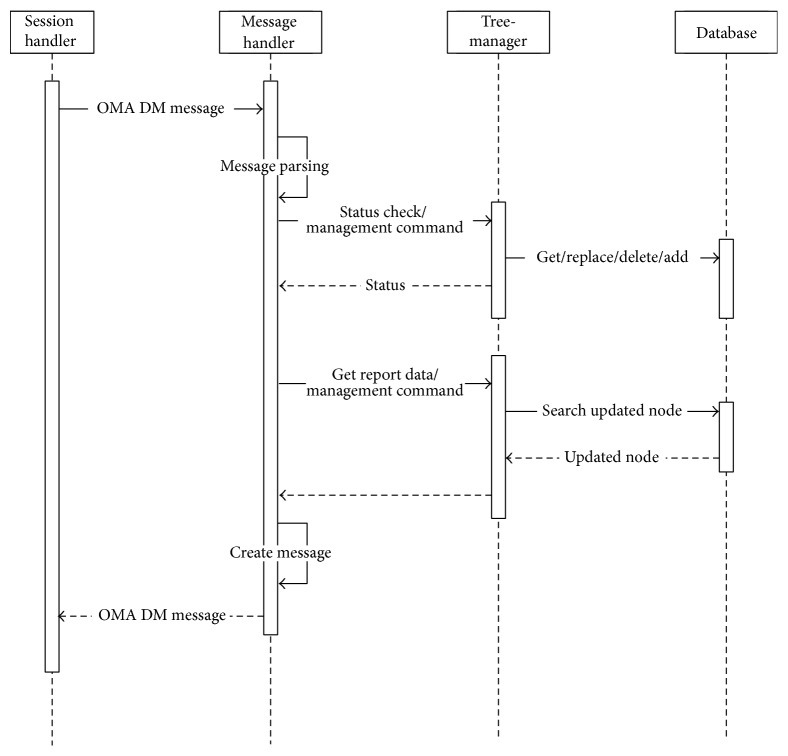
Sequence diagram of messages in a DM gateway.

Figure 12 shows the sequence diagram of messages in a DM gateway.

### 3.5. Multilayered Health Management Server

**Figure 13:**
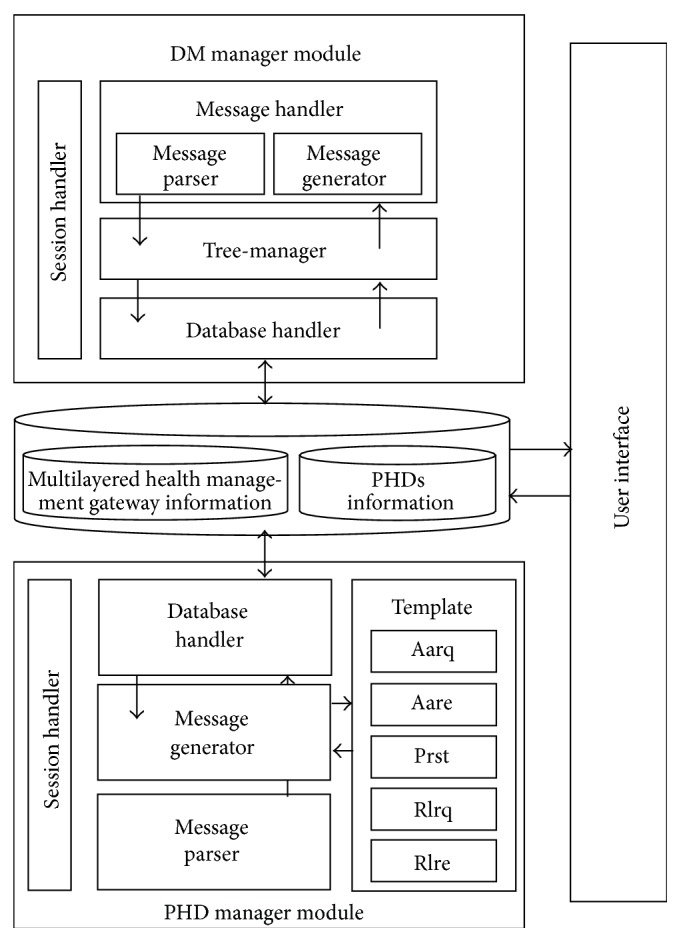
Structure of the multilayered health management server.

Figure 13 shows the structure of the multilayered health management server. Medical personnel or system managers monitor a PHD user's health information, issue remote management commands, and receive reports through the server. The components of the server are very similar to those of the gateway, and thus an explanation of these will be omitted.

**Figure 14:**
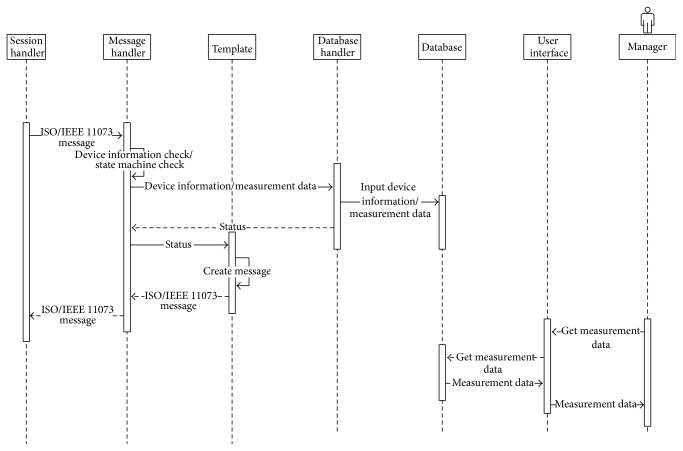
Sequence diagrams in a PHD manager module in the server.

**Figure 15:**
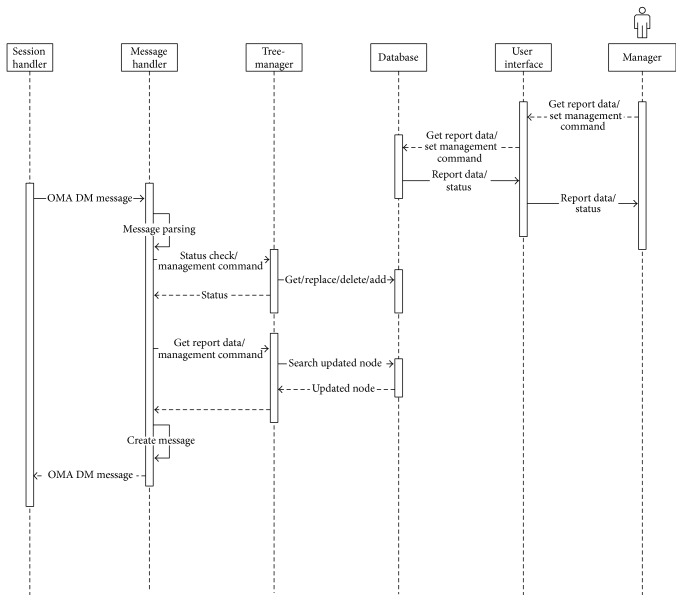
Sequence diagrams in a DM manager module in the server.

Figures 14 and 15 show the sequence diagrams in a PHD manager module and a DM manager module in the server, respectively.

## 4. Security Considerations

### 4.1. Biomedical Signature

Whenever biomedical data is processed, security issues related to the data inevitably arise. In this paper, the concept of the biomedical signature is proposed to address this issue. The biomedical signature is used to authenticate users as well as messages transmitted between a PHD and a monitoring server. The biomedical status of a user is characterized by the user's biomedical signature. The biomedical signature of a user is a hash value calculated from the highest values and the lowest values of the biomedical data, which is obtained by the PHDs the user carries for a certain period of time.

In other words, BM-signature (*n*,* t*), the biomedical signature of user* n* during most the recent* t* months, is defined as follows.

BM-signature (*n*,* t*) = hash_auth(a string of (Hbp, Lbp, Hgc, Lgc,…, Hk, Lk)), where hash_auth is a hash function for authentication. Hk (Lk) is the highest (the lowest) value of* k*-type biomedical data of user* n* measured in the recent* t* months using his/her PHD, while bp and gc represent blood pressure and glucose, respectively. For example, let us assume that the highest and the lowest values of biomedical data of user* n* during the last one month are as follows.

The highest (lowest) values of blood pressure, glucose, pulse rate, and body fat percentage are 120 (80), 110 (70), 100 (60), and 20 (15), respectively. SHA512 [18] is assumed to be used for a hash function.

Then BM-signature (*n*, 1) = SHA512(120, 80, 110, 70, 100, 60, 20, 15).

Then, the BM-signature proposed in this paper for authentication has the following properties:

- Since the possibility of having the same biomedical data between any two users measured during the recent time interval is very low, BM-signature (*n*,* t*) is almost unique and represents very well the characteristics of the biomedical data of user* n* during the recent* t* months. It can be said that as the number of biomedical data used for authentication increases, the degree of uniqueness increases.
- Since BM-signature (*n*,* t*) varies as time changes, BM-signature (*n*,* t*) may have the time stamp property [18], which is considered one of the essential properties to defend against active attacks. As* t* increases, the BM-signature acquires a more perfect time stamp property.

### 4.2. Authentication

**Figure 16:**
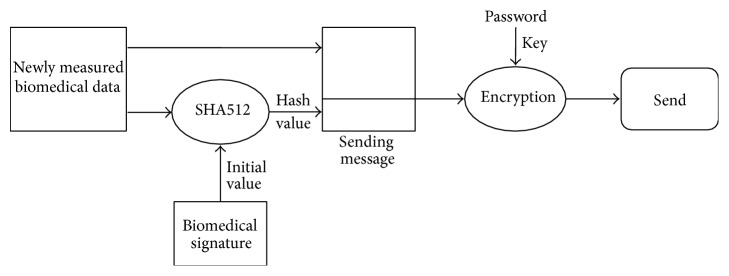
Authentication process for sender side.

**Figure 17:**
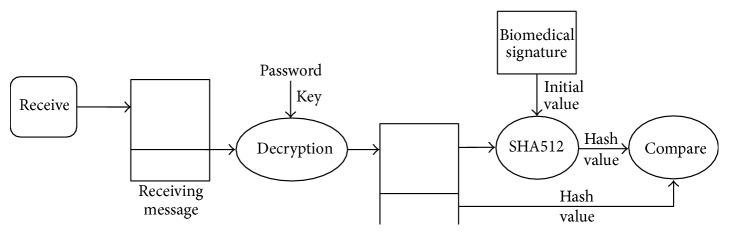
Authentication process for receiver side.

The biomedical data of a user must be securely protected and cannot be accessed/altered by an unauthorized person, as this can cause a serious invasion of privacy. For this reason, the system proposed in this paper provides user/message authentication processes to securely transmit the biomedical data measured by PHDs. Figures 16 and 17 show the authentication processes for the sender side and receiver side, respectively. In Figure 16, new biomedical data of a user (sender) measured by a PHD is given to the SHA512 hash algorithm to produce a 512-bit hash value that will be used to authenticate the new biomedical data by the receiver (the health management server). Since the SHA512 algorithm needs an initial value to be executed, the BM-signature of the user is used as the initial value. Thus, a message sent consists of the new biomedical data and the hash value of the data. Finally, the message is encrypted with the user password as the encryption key before it is sent. DES (Data Encryption Standard) encryption algorithm [18] is used in this paper.

Upon receiving the message sent by the sender (user), the receiver authenticates the received message, as shown in Figure 17. First of all, the received message is decrypted by using the DES decryption algorithm with the decryption key (user password), which is the same as the encryption key, in order to get the biomedical data and its hash value from the message. Next, the receiver executes the SHA512 algorithm to produce the message's hash value in order to compare the newly calculated hash value by the receiver with the hash value contained in the received message. If the two hash values are the same, the receiver decides that the received message has been authenticated. In other words, the receiver is sure that the receiving message is sent by the user who claims to be the sender and that the message has been unaltered during transmission. If the two have values that are not the same, the authentication fails and the receiver rejects the message.

## 5. Results and Discussion

### 5.1. Experiment Environments

The secure biomedical data management system explained above is constructed and tested in the experiment environments shown in Table 1. The executable code sizes of the PHD agent and DM agent are 439 KB and 179 KB, respectively, which indicates the PHD used in this study has sufficient room for two embedded agents.

### 5.2. Experiments

Some experiments are performed to show that the system proposed in this study works as designed. Because it is assumed that the system proposed in this study has a very large number of PHDs, a stress test is needed to make sure that the system is stable in heavy loaded environments. For the experiments, a 4-layered system (including 2 layers of gateways) is constructed. For a stress test, up to 1,200 threads are made to represent the same number of PHD agents. In addition, it is necessary to find the bottlenecks to improve the overall system performance.

**Figure 18:**
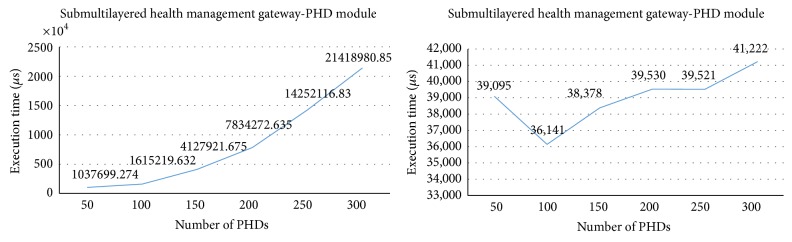
Execution times in the subgateway.

Figure 18 shows the execution times in the subgateway (i.e., the gateway in the lower layer) as the number of PHD agents increase. The subgateway is connected to up to three hundred PHD agents. The execution time in the PHD module of the subgateway increases exponentially as the number of PHD agents increases, while the execution time in the OMA DM module remains practically unchanged. This is because the structures of the databases that store the health data (of PHD module) and the management data (of DM module) are different.

**Figure 19:**
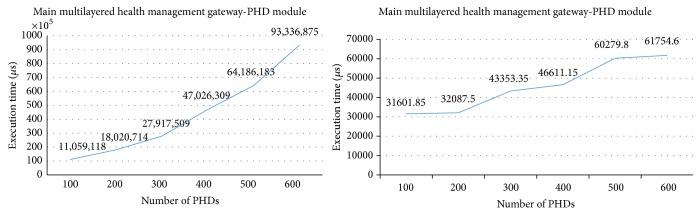
Execution times in the main gateway.

Figure 19 shows the execution times in the main gateway (i.e., the gateway in the higher layer) as the number of PHD agents increases. The main gateway is connected to two subgateways which connect to up to three hundred PHD agents each, having up to six hundred PHD agents connecting to the main gateway. The execution time in the PHD module of the main gateway increases exponentially as the number of PHD agents increases, while the execution time in the OMA DM module increases proportionally.

**Figure 20:**
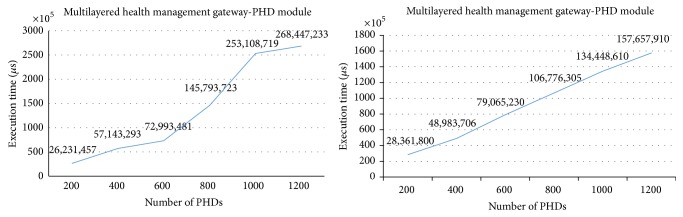
Execution times in the server.

Figure 20 shows the execution times in the server as the number of PHD agents increases. The server is connected to two main gateways that connect to up to six hundred PHD agents each, having up to 1,200 PHD agents connecting to the main gateway. The execution time in the PHD module of the server increases exponentially as the number of PHD agents increases, while the execution time in the OMA DM module increases proportionally.

**Figure 21:**
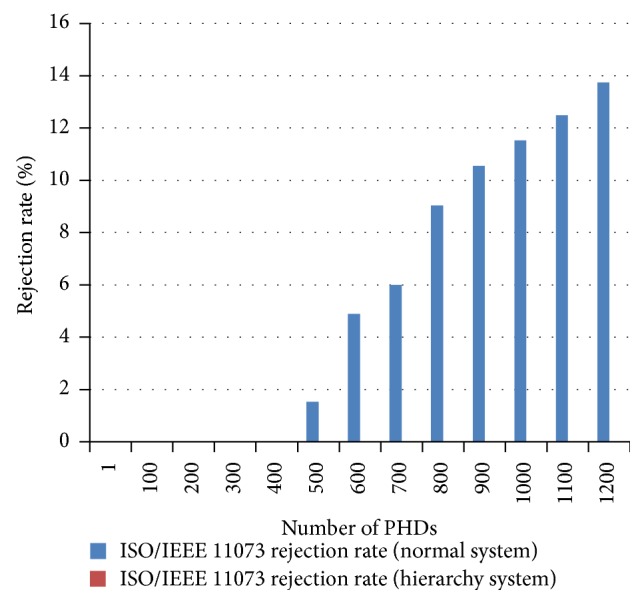
ISO/IEEE 11073 message loss ratios by the server.

Because the ISO/IEEE 11073 messages are much more sensitive than the OMA DM messages to the number of PHD agents connected at once, one more experiment is performed to find out how many ISO/IEEE 11073 messages are lost at the server. When communication traffic becomes heavy, the number of messages that arrive but are rejected by the server becomes large. Loss of messages is considered a serious matter in health management systems.  Figure 21 shows the ISO/IEEE 11073 message loss ratios by the server as the number of PHD agents increases. The loss of ISO/IEEE 11073 messages occurs when more than 500 PHDs are connected at the same time in the normal system (i.e., without any gateways at all). The loss ratio of the ISO/IEEE 11073 messages in the normal system is as high as 14% when 1,200 PHD agents are connected. On the other hand, no message loss occurs in the multilayered system proposed in this study, which demonstrates the superiority of the multilayered system to the normal system with regard to heavy traffic.

### 5.3. Performance Improvement

#### 5.3.1. Bottlenecks

An attempt was made to find bottlenecks in the system constructed in this study in order to improve the performance of the system.

- PHD gateway module in the gateway and the server: almost 80% of the execution time in the gateway and the server is spent accessing/updating their own databases. Because the databases used in this study do not support parallel database operations, all of database requests must be executed in a critical section, which means that all of the requests are executed sequentially.
- DM gateway module in the gateway and the server: it is found that almost 90% of the execution time in the gateway and the server is spent updating their DM trees. Unlike the PHD module, the DM module uses a hierarchical database based on XML files, which is thought to be the appropriate database type to store DM trees. As a result, execution times for tree searching and updating take up most of the execution times in the gateway and the server.

#### 5.3.2. Bottleneck Alleviation

*(1) Bottleneck Alleviation at PHD Gateway and the Server*. The system uses four databases: DataInfo, DataReportingInfo, DeviceInfo, and Mapping. Among the databases, it is found that the execution time for accessing/updating DataReportingInfo database is the longest. The database keeps health data measured by PHDs. Therefore, in this study, the system is modified in such a way that the DataReportingInfo database is divided into as many PHD databases as the number of PHD types used (three in the experiment). Likewise, databases for the subgateways and the main gateway are also divided according to the number of subgateways connecting to the main gateway.

**Figure 22:**
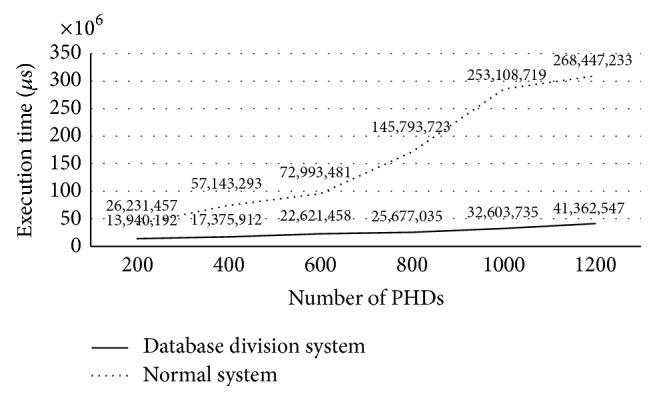
Performance improvement by the database division scheme.

Figure 22 shows the average execution times at the server for ISO/IEEE 11073 messages. The dotted line in the graph represents the waiting times at the server with one DataReportingInfo database, as was explained earlier. The solid line represents the execution times at the server, with DataReportingInfo database divided into three. As shown in the figure, the database division scheme greatly improves the system performance when the database is divided according to the connected PHD types. The relative performance improvement ratios with 200 and 1,200 PHDs areas are as high as 46.85% and 84.59%, respectively, 73.25% on average in the graph.

*(2) Bottleneck Alleviation at DM Gateway and the Server*. This study uses the delayed write scheme to reduce the average execution times for tree searching and updating operations in the gateway and the server. To reduce the DM tree searching time, a hash table is constructed to eliminate frequent I/O interrupts, instead of direct access to the DM trees. In addition, the XML database is cached by the tree-manager module. In this experiment, the delayed write operation of the cached XML database is performed every 10 minutes or every time the number of connected PHDs at the same time falls below one hundred.

**Figure 23:**
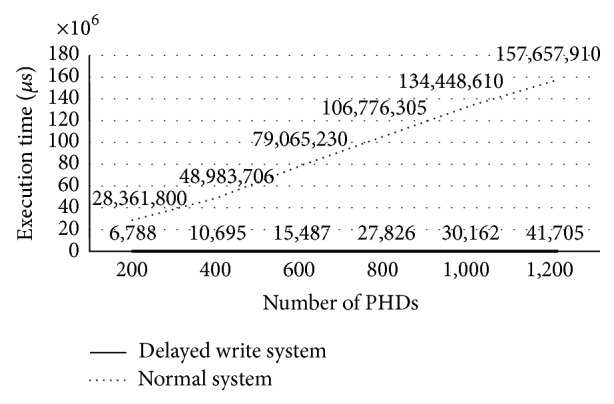
Performance improvement by the delayed write scheme.

Figure 23 shows the average execution times at the server for OMA DM messages. The dotted line in the graph represents the execution times at the server without the delayed write operation, while the solid line represents the execution times at the server with the cached DM trees and the delayed write operation. As shown in the figure, the delayed write scheme greatly improves the system performance when there is a delayed write operation of the cached DM trees. The relative performance improvement ratio is as high as 99.99%, on average, in the graph.

## 6. Conclusions and Future Research

In this study, a multilayered remote PHD management system for a very large number of PHDs is proposed. The system has the following characteristics: first, the system is designed in a hierarchical fashion to lessen the bottleneck problem which might be caused by the requests from a very large number of PHDs. For the experiments explained earlier, a 4-layered hierarchical system including 2 layers of gateways is constructed. Second, the system is proposed to manage diverse PHDs remotely. The system has a separate database for every type of PHD to shorten database access times. Third, the system supports international standard communication protocols to achieve interoperability. Two protocols, the ISO/IEEE 11073 protocol and the OMA DM protocol, which were proposed as international standard communication protocols for PHD communication and remote device management, respectively, are extended and implemented in the system. Fourth, the system is integrated in the sense that both a PHD communication system and a remote PHD management system work together as a single system. Gateways between PHDs and servers are designed to handle the integration tasks in the system.

Some experiments, including the stress test, are carried out to show that the system proposed in this study performs very well even when a very large number of PHDs are used. After locating the bottlenecks, it is found that there is still room to improve the system performance, and two modifications of the system were performed in this study: database division and delayed write. It is found that the system performance is greatly improved after these two modifications are applied.

Moreover, message loss is considered to be a very serious matter in health related systems such as the one outlined in this study. From the experiments, it is found that the loss ratio of ISO/IEEE 11073 messages in the normal system (e.g., the system without any gateways) is quite high when there is heavy traffic, while no message loss occurs in the multilayered system proposed in this study, demonstrating the superiority of the multilayered system to the normal system in situations of heavy traffic.

However, the multilayered system is not completely flawless. One flaw that the system might harbor is poor fault-tolerance. When some of the gateways fail, more complicated recovery schemes are needed. Therefore, efficient recovery/backup schemes or protocols will be studied to enhance the fault-tolerance of the system.

## Figures and Tables

**Table 1:** Experiment environments.

Category	Multilayered health management server	Multilayered health management gateways	PHD
CPU	Intel core i7-3770 (3.4 Ghz)	Intel core i5-650 (3.2 Ghz)	Intel core i3-2367M (1.4 Ghz)

Main memory	8 GB	4 GB	2 GB

Hard disk type	SSD	SSD	HDD

Operating system	Windows 7	Windows 7	Windows 7

Language	C#	C#	C#
